# Temporal and Spatial Dynamics of Archaeal Communities in Two Freshwater Lakes at Different Trophic Status

**DOI:** 10.3389/fmicb.2016.00451

**Published:** 2016-03-31

**Authors:** Yuyin Yang, Yu Dai, Zhen Wu, Shuguang Xie, Yong Liu

**Affiliations:** ^1^State Key Joint Laboratory of Environmental Simulation and Pollution Control, College of Environmental Sciences and Engineering, Peking UniversityBeijing, China; ^2^Key Laboratory of Water and Sediment Sciences, Ministry of Education, College of Environmental Sciences and Engineering, Peking UniversityBeijing, China

**Keywords:** freshwater lake, microbial community, *Euryarchaeota*, planktonic, sediment, *Bathyarchaeota*, trophic status

## Abstract

In either eutrophic Dianchi Lake or mesotrophic Erhai Lake, the abundance, diversity, and structure of archaeaplankton communities in spring were different from those in summer. In summer, archaeaplankton abundance generally decreased in Dianchi Lake but increased in Erhai Lake, while archaeaplankton diversity increased in both lakes. These two lakes had distinct archaeaplankton community structure. Archaeaplankton abundance was influenced by organic content, while trophic status determined archaeaplankton diversity and structure. Moreover, in summer, lake sediment archaeal abundance considerably decreased. Sediment archaeal abundance showed a remarkable spatial change in spring but only a slight one in summer. The evident spatial change of sediment archaeal diversity occurred in both seasons. In Dianchi Lake, sediment archaeal community structure in summer was remarkably different from that in spring. Compared to Erhai Lake, Dianchi Lake had relatively high sediment archaeal abundance but low diversity. These two lakes differed remarkably in sediment archaeal community structure. Trophic status determined sediment archaeal abundance, diversity and structure. Archaeal diversity in sediment was much higher than that in water. Water and sediment habitats differed greatly in archaeal community structure. *Euryarchaeota* predominated in water column, but showed much lower proportion in sediment. *Bathyarchaeota* was an important component of sediment archaeal community.

## Introduction

*Archaea* might participate in biogeochemical cycling of carbon, nitrogen and sulfur (Hu et al., 2015; Zhang et al., 2015). Planktonic archaeal populations can be an important component of prokaryotic community in freshwater lakes (Lehours et al., 2005; Auguet and Casamayor, 2008; Callieri et al., 2009), but their abundance and structure can vary considerably with both time and water depth (Pernthaler et al., 1998; Casamayor et al., 2000, 2001; Keough et al., 2003; Lehours et al., 2005, 2007; Callieri et al., 2009; Berdjeb et al., 2013; Vila-Costa et al., 2013; Li et al., 2015). Archaeaplankton communities in different lacustrine ecosystems also show the marked dissimilarity (Casamayor et al., 2000, 2001; Keough et al., 2003; Auguet and Casamayor, 2008; Vila-Costa et al., 2013). However, information on the horizontal change of archaeaplankton community in freshwater lake is still limited (Keough et al., 2003; Li et al., 2015). So far, the links between environmental variables and archaeaplankton community remain not well understood. A number of environmental factors might collectively regulate freshwater lake archaeaplankton community (Berdjeb et al., 2013).

Recently, the distribution of sediment archaeal community in freshwater lake has received increasing attention. The horizontal and vertical changes of sediment archaeal community in freshwater lake ecosystem have been well-documented (Liu et al., 2009, 2010; Ye et al., 2009; Haller et al., 2011; Bhattarai et al., 2012; Borrel et al., 2012; Billard et al., 2015; Chen et al., 2015), yet the seasonal effect on sediment archaeal community remains under debate. For example, Rodrigues et al. (2014) revealed the profound seasonal effect on sediment archaeal community structure in a Cerrado lake, whereas a slight seasonal shift in sediment archaeal community occurred in other freshwater lakes (Schwarz et al., 2007; Chen et al., 2015). Information on the comparison of sediment archaeal communities in different lacustrine ecosystems is still very limited. Only a recent study showed a large discrepancy of archaeal community abundance and structure in profundal sediments of different freshwater lakes on the Yunnan Plateau (Zhang et al., 2015). To date, the environmental factors driving the spatiotemporal dynamics of lake sediment archaeal community remain essentially unclear. Moreover, there has been no report available on the difference between planktonic and sediment archaeal communities in freshwater lake. Therefore, the main goal of this current study was to investigate the spatial and temporal dynamics of both planktonic and sediment archaeal populations in freshwater lake and the associated environmental factors. The discrepancy of planktonic and sediment archaeal communities was also investigated.

## Materials and Methods

### Study Sites and Sampling

Eutrophic Dianchi Lake (309 km^2^) and mesotrophic Erhai Lake (250 km^2^) are the largest two freshwater lakes on the Yunnan Plateau (China). The average water depth of Dianchi Lake and Erhai Lake were 4.4 and 10 m, respectively (Wang et al., 2015). In both April (spring, dry season) and August (summer, rainy season) in 2015, triplicate water samples (30 cm depth below water surface, about 10 L) and sediment cores (about 2 kg) were collected from six different sampling locations in either Dianchi Lake (D1–D6) or Erhai Lake (E1–E6) (Supplementary Figure S1), using plexiglass water sampler and Kajak tube core sampler (Denmark), respectively. The sediment cores were sliced into layers, and the upper layer (0–10 cm) was used for further chemical and molecular analyses. The physicochemical properties of lake water and sediment samples were shown in Supplementary Figures S2 and S3, respectively (Dai et al., 2015). These water and sediment samples had been used for the molecular analysis of bacterial communities in our previous study (Dai et al., 2015).

### Quantitative PCR Analysis

For molecular analysis, water samples (300 mL) were prefiltered through a 40-μm pore-size net, and subsequently microbial cells in waters was retained using 0.22-μm pore-size membrane (diameter 50 mm; Millipore). Genomic DNA of the retained biomass was extracted using E.Z.N.A. Water DNA kit (Omega, USA). Lake sediment genomic DNA was extracted using Powersoil DNA extraction kit (Mobio Laboratories, USA). The quality of DNA were checked by 1.0% agarose gel electrophoresis and quantified using a biophotometer (Eppendorf, Hamburg, Germany). The number of archaeal 16S rRNA gene was quantified by real-time quantitative PCR (q-PCR) using the primer sets Arch349F (5′-GYGCASCAGKCGMGAAW-3′)/Arch806R (5′-GGACTACVSGGGTATCTAAT-3′) (Jung et al., 2011; Liu et al., 2014). SYBR Green q-PCR was carried out using an ABI 7500 FAST (Applied Biosystems). The 25-μL reaction mixture included 2× SYBR Green PCR master mix (12.5 μL), 10 μM Arch349F and Arch806R primers (1 μl), and template DNA (2 ng). The amplification conditions were as follows: 10 min at 95°C followed by 40 cycles of 95°C for 15 s, annealing for 60 s at 60°C, and extension at 72°C for 30 s. Melting curve analysis was carried out from 60 to 95°C. Standard curves ranging from 10^1^ to 10^8^ gene copies/mL were obtained using serial dilutions of linearized plasmids (pGEM-T, Promega) containing cloned 16S rRNA genes amplified from environmental DNA. The average amplification efficiency and coefficient (*r^2^*) for archaeal 16S rRNA gene were 96 % and 0.998, respectively.

### High-Throughput Sequencing Analysis

In this study, the total genomic DNA of each water or sediment sample was amplified using the archaeal primers Arch519F (5′-CAGCCGCCGCGGTAA-3′)/Arch915R (5′-GTGCTCCCCCGC CAATTCCT-3′) following the same PCR conditions as previously described (Coolen et al., 2004). Amplicons were verified by 1% agarose gel electrophoresis and purified using QIAquick PCR purification kit (Qiagen Inc.). The PCR products from triplicate samples were mixed in equal amounts and then were subject to Illumina MiSeq high-throughput sequencing at Shanghai Majorbio Bio-pharm Technology Co., Ltd (China). The obtained raw reads were deposited in the NCBI short-read archive under accession numbers SRP067264 (water samples) and SRP067256 (sediment samples). The raw Illumina reads were merged using FLASH and further processed following the protocol (Caporaso et al., 2010). The chimeric reads were discarded using UCHIME (Edgar et al., 2011). Chimeric-free sequences sharing ≥7% similarity were clustered into operational taxonomic units (OTUs), and then Chao1 richness estimator and Shannon and Simpson indices were generated using the UPARSE pipeline (Edgar, 2013). To examine the difference in the overall community composition between each pair of samples at the OTU level, the beta diversity analysis was performed using UniFrac (Lozupone and Knight, 2005). Unweighted UniFrac with the Quantitative Insights into Microbial Ecology (QIIME) program was applied for unweighted pair group method with arithmetic mean (UPGMA) clustering. In addition, the taxonomic identities (at phylum and class levels) of archaeal sequences were classified using the Silva 16S rRNA database (Quast et al., 2013) and were further validated by phylogenetic analyses using MEGA software version 6.0 (Tamura et al., 2013).

### Statistical Analysis

One-way analysis of variance (ANOVA) followed by Student–Newman–Keuls test was applied to determine the significant difference (*P* < 0.05) in the density of archaeal 16S rRNA gene among samples. The potential links between the determined physicochemical properties and the abundance, richness and diversity of archaeal populations or the proportion of the dominant archaeal groups were examined with Spearman rank correlation analysis using the software SPSS 20.0. To explore the correlations between overall archaeal community composition (OTU level) and the environmental factors, detrended correspondence analysis (DCA) was used to select the suitable ordination analysis method. The longest DCA axis was found to have a gradient length less than 3 SD units, so redundancy analysis (RDA) was performed (Lepš and Šmilauer, 2003) with Monte Carlo tests using CANOCO 4.5 software (Biometrics Wageningen, The Netherlands). In this study, the number of sequences in each major archaeal OTU (defined at 50 sequences threshold) was assigned as species input, whereas water or sediment physicochemical property was used as environmental input (Zhang et al., 2015).

## Results

### Archaeal Community Abundance

**FIGURE 1:**
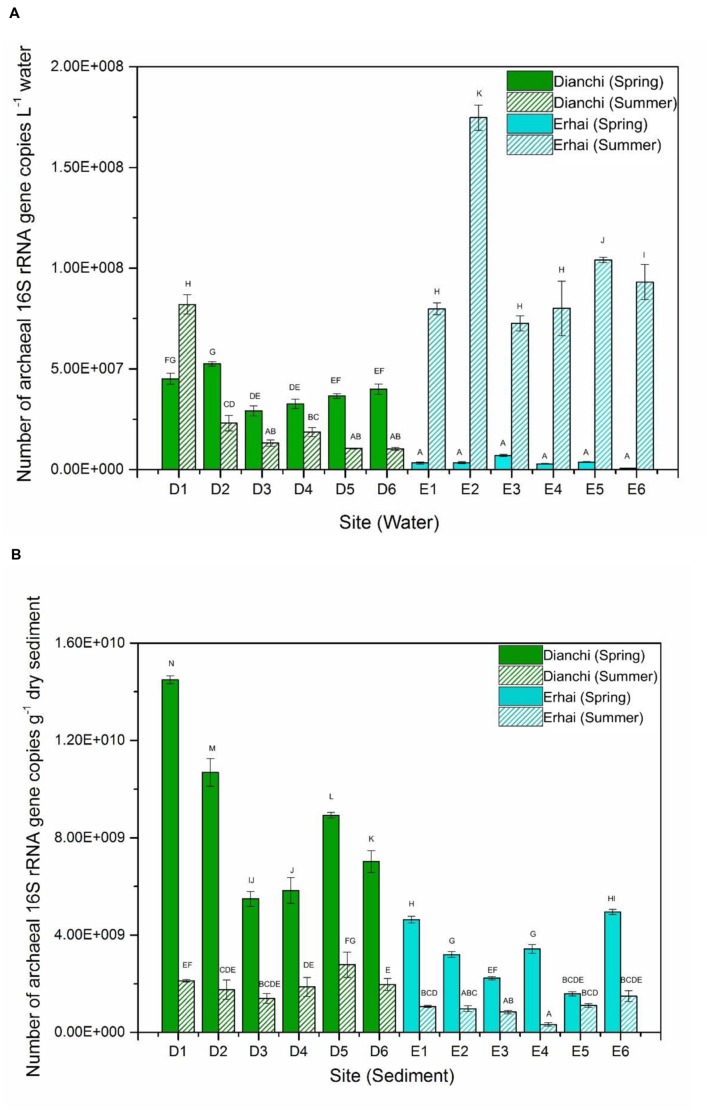
**Abundance of archaeal 16S rRNA gene in spring and summer water samples (A) and sediment samples (B) from different sampling locations in Dianchi Lake and Erhai Lake.** Different letters above the columns indicate the significant differences (*P* < 0.05).

The density of planktonic archaeal 16S rRNA gene ranged from 1.03 ± 0.07 × 10^7^ to 8.2 ± 0.48 × 10^7^ copies per L water in Dianchi Lake, whereas waters in Erhai Lake had the archaeal community size of 6.75 ± 1.71 × 10^5^ to 1.75 ± 0.06 × 10^8^ 16S rRNA gene copies per L water (**Figure 1A**). In spring, archaeaplankton abundance at sampling site D2 significantly outnumbered that at sampling sites D3, D4, D5 and D6 (*P* < 0.05), and the significant difference in archaeaplankton abundance was also observed between sites D1 and D3 (or D4). In summer, archaeaplankton abundance at site D1 was significantly higher than that at other five sites (*P* < 0.05), and archaeaplankton was more abundant at site D2 than at sites D3, D5, and D6 (*P* < 0.05). These results displayed the considerable spatial fluctuation of archaeaplankton abundance in Dianchi Lake in both spring and summer. At a given sampling site in Dianchi Lake, the significant difference in archaeaplankton abundance could be observed between spring and summer water samples (*P* < 0.05). The summer water sample generally had much lower archaeaplankton abundance than the corresponding spring one. Moreover, the six spring waters from Erhai Lake illustrated no significant difference in archaeaplankton abundance (*P*> 0.05), suggesting the relatively slight spatial shift in archaeaplankton abundance. However, in summer, archaeaplankton abundance at sites E1, E3, and E4 was significantly lower than that at other three sites (*P* < 0.05), illustrating the remarkable spatial variation of archaeaplankton abundance. At a given sampling site in Erhai Lake, archaeaplankton abundance considerably increased in summer. In addition, Dianchi Lake had higher archaeaplankton abundance than Erhai Lake in spring, while an opposite trend was found in summer.

The number of archaeal 16S rRNA gene in sediments of Dianchi Lake ranged between 1.4 ± 0.2 × 10^9^ and 1.45 ± 0.02 × 10^10^ copies per gram dry sediment, while sediments of Erhai Lake showed the archaeal abundance of 3.3 ± 0.65 × 10^8^–4.95 ± 0.11 × 10^9^ 16S rRNA gene copies per gram dry sediment (**Figure 1B**). In spring, the sediment archaeal abundance at one sampling site in Dianchi Lake was generally different from that at other sampling sites (*P* < 0.05), suggesting the remarkable spatial change of sediment archaeal abundance. In contrast, no significant difference in archaeal abundance was found among the summer sediments at sites D1, D2, D3, D4, and D6, indicating the relatively slight spatial variation of sediment archaeal abundance. At a given sampling site in Dianchi Lake, sediment archaeal abundance considerably decreased in summer. Moreover, in spring, sediments at sites E1 and E6 had significantly higher archaeal abundance than those at other four sites (*P* < 0.05), and sediments at sites E2 and E4 also showed significantly higher archaeal abundance than those at sites E3 and E5 (*P* < 0.05). This showed the remarkable spatial fluctuation of archaeal abundance in Erhai Lake spring sediments. However, no significant difference in archaeal abundance was observed among the summer sediments at sites E1, E2, E3, E5, and E6 (*P*> 0.05), indicating the relatively slight spatial variation of sediment archaeal abundance in summer. At a given sampling site in Erhai Lake, sediment archaeal abundance generally decreased in summer. In addition, in either spring or summer, Dianchi Lake tended to have higher sediment archaeal abundance than Erhai Lake.

### Archaeal Community Richness and Diversity

**FIGURE 2:**
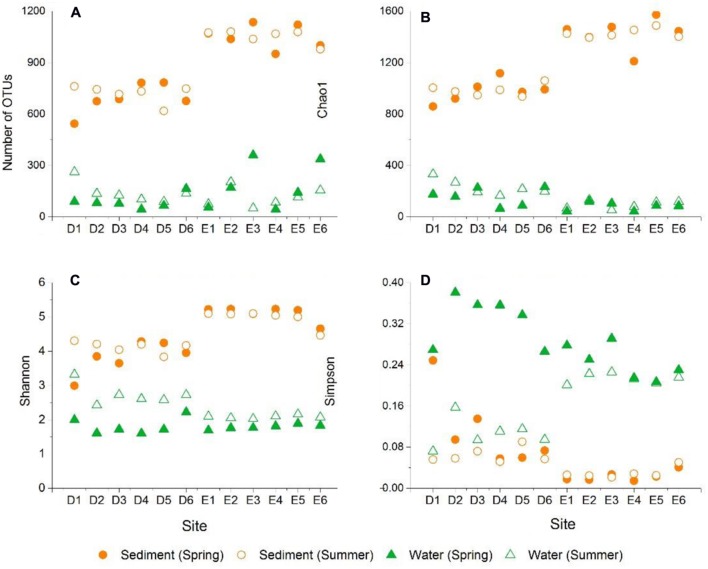
**Archaeal community operational taxonomic units (OTUs) (A), Chao1 richness estimator (B), Shannon index (C), and Simpson index (D)**.

**FIGURE 3:**
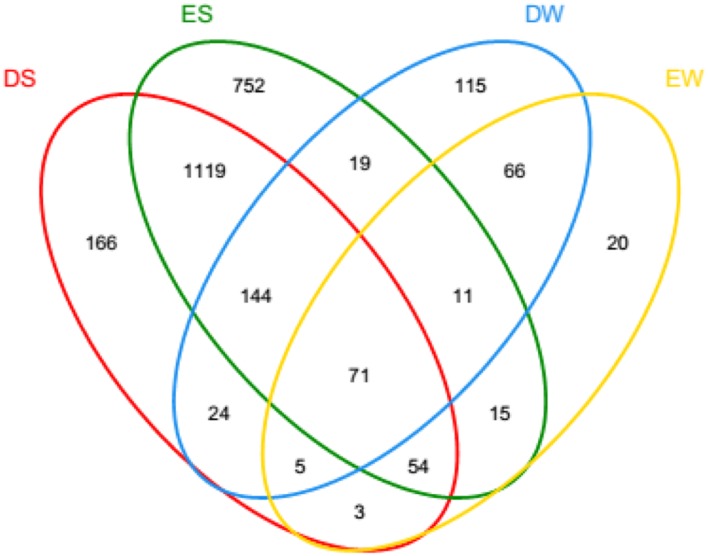
**Share OTUs among waters and sediments for pooled sequences.** The Venn diagram was plotted using program *R*. DS and ES represent the sediments from Dianchi Lake and Erhai Lake, respectively, while DW and EW represent the waters from Dianchi Lake and Erhai Lake, respectively.

In the current study, the number of high-quality archaeal sequences from each lake sample was normalized to 17,319 for the comparison of community richness and diversity. High Good’s coverage (≥98%) illustrated that the OTUs of each water or sediment archaeal library had been well captured. All of the normalized water and sediment archaeal sequences could be grouped into 2,600 OTUs. Dianchi Lake waters comprised 44–262 archaeal OTUs, while Erhai Lake waters consisted of 44–360 archaeal OTUs (**Figure 2A**). An obvious spatial change of bacterioplankton OTU number occurred in these two freshwater lakes. At a given sampling site in Dianchi Lake, the summer water sample generally had more OTUs than the corresponding spring one. Moreover, a total of 26 major OTUs (with relative abundance of no less 1% in at least one water sample) were detected in lake waters (Supplementary Table S1), while 36 major OTUs (with relative abundance of no less 1% in at least one sediment sample) were detected in lake sediments (Supplementary Table S2). The changes of the proportions of the major OTUs with sampling site and time were found in either Dianchi Lake or Erhai Lake. Venn diagrams demonstrate the strong overlap in OTUs between Dianchi Lake and Erhai Lake sediments, while only 33.6% of OTUs in Dianchi Lake water was identified in Erhai Lake water (**Figure 3**). In addition, 15.4% of OTUs in Dianchi Lake sediment was identified in Dianchi Lake water, and only 6.9% of OTUs in Erhai Lake sediment was present in Erhai Lake water.

The Chao1 richness estimators of planktonic archaeal communities in Dianchi Lake and Erhai Lake were 64–331 and 42–132, respectively (**Figure 2B**), showing an evident spatial change of archaeaplankton richness. In summer, Dianchi Lake had higher archaeaplankton richness than Erhai Lake. Moreover, sediments had much higher OTUs and Chao1 richness than waters. Sediments in Dianchi Lake had 543–782 OTUs and the Chao1 estimator of 858–1136, while sediments in Erhai Lake showed more OTUs (950–1135) and higher Chao1 estimator (1210–1572). In either spring or summer, the evident spatial change of sediment archaeal OTU number and Chao1 richness was found in these two lakes.

Archaeaplankton communities in Dianchi Lake and Erhai Lake showed the Shannon diversity indices of 1.6–3.32 and 1.69–2.16, respectively (**Figure 2C**). In summer, Dianchi Lake had higher archaeaplankton community diversity than Erhai Lake.

In either spring or summer, archaeaplankton diversity in Dianchi Lake showed a remarkable spatial variation, while only a slight spatial variation occurred in Erhai Lake. At a given sampling site in either of these two lakes, archaeaplankton diversity was relatively high in summer. Moreover, lake sediments generally had much higher archaeal diversity than waters. Sediments in Dianchi Lake had archaeal Shannon diversity indices of 2.99–4.3, while those in Erhai Lake had relatively higher diversity (4.46–5.23). In either spring or summer, sediment archaeal diversity in both of these two lakes showed a remarkable spatial variation. At a given sampling site in Erhai Lake, sediment archaeal diversity slightly increased in summer.

Simpson indices of planktonic and sediment archaeal communities were 0.07–0.38 and 0.01–0.25, respectively (**Figure 2D**). In either spring or summer, planktonic archaeal community evenness in both Dianchi Lake and Erhai Lake showed a remarkable spatial variation. At a given sampling site in either of these two lakes, archaeaplankton community evenness decreased in summer. Lake sediments generally had lower archaeal evenness than waters. The spatial variation of sediment archaeal evenness in Dianchi Lake was larger than that in Erhai Lake. Sediment archaeal evenness in Dianchi Lake generally decreased in summer, but an opposite trend was found in Erhai Lake.

### UPGMA Clustering Analysis of Archaeal Communities

**FIGURE 4:**
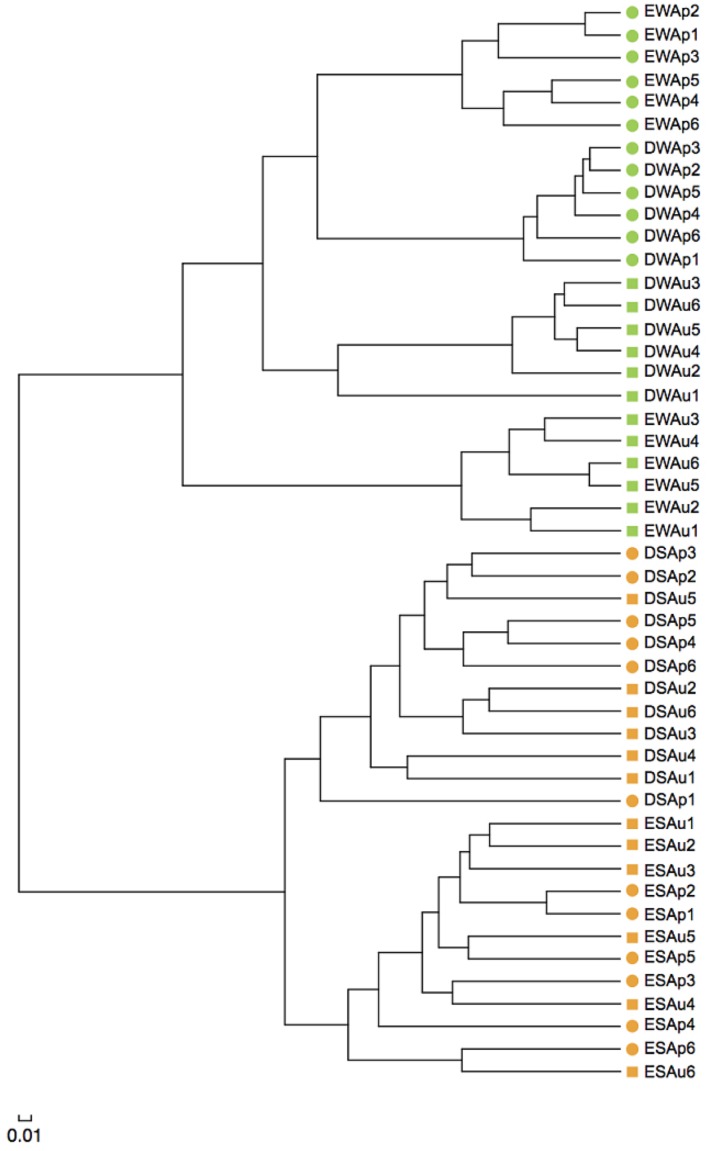
**Unweighted pair group method with arithmetic mean (UPGMA) clustering of water and sediment samples from Dianchi Lake and Erhai Lake.** Samples DWAp1, DWAp2, DWAp3, DWAp4, DWAp5, and DWAp6 represent the April water samples from sites D1–D6 in Dianchi Lake, respectively. Samples DWAu1, DWAu2, DWAu3, DWAu4, DWAu5, and DWAu6 represent the August water samples from sites D1–D6 in Dianchi Lake, respectively. Samples EWAp1, EWAp2, EWAp3, EWAp4, EWAp5, and EWAp6 represent the April water samples from sites E1–E6 in Erhai Lake, respectively. Samples EWAu1, EWAu2, EWAu3, EWAu4, EWAu5, and EWAu6 represent the August water samples from sites E1–E6 in Erhai Lake, respectively. Samples DSAp1, DSAp2, DSAp3, DSAp4, DSAp5, and DSAp6 represent the April sediment samples from sites D1–D6 in Dianchi Lake, respectively. Samples DSAu1, DSAu2, DSAu3, DSAu4, DSAu5, and DSAu6 represent the August sediment samples from sites D1–D6 in Dianchi Lake, respectively. Samples ESAp1, ESAp2, ESAp3, ESAp4, ESAp5, and ESAp6 represent the April sediment samples from sites E1–E6 in Erhai Lake, respectively. Samples ESAu1, ESAu2, ESAu3, ESAu4, ESAu5, and ESAu6 represent the August sediment samples from sites E1–E6 in Erhai Lake, respectively.

The result of UPGMA clustering showed that water samples were clearly separated from sediment samples, illustrating the distinct structure difference between planktonic and sediment archaeal communities (**Figure 4**). Water samples could be further separated into four distinct clades. In either spring or summer, Dianchi Lake waters could be clearly separated from Erhai Lake waters. For either Dianchi Lake or Erhai Lake, spring waters were clearly separated from summer waters, suggesting that archaeaplankton community in summer was remarkably different from that in spring. Moreover, Dianchi Lake sediment samples were distantly separated from Erhai Lake sediment samples, suggesting the distinct structure difference of sediment archaeal communities between in these two lakes. At a given sampling site in Dianchi Lake, the spring and summer sediment samples were not closely clustered together, indicating sediment archaeal community in summer was remarkably different from that in spring. However, at sampling sites E1, E2, E5, or E6, the spring and summer sediment samples tended to be grouped together, suggesting the relatively slight temporal variation of sediment archaeal community structure in Erhai Lake.

### Archaeal Community Composition

In the present study, phylum *Euryarchaeota* predominated in each of the water samples from Dianchi Lake and Erhai Lake (accounting for ≥99.9%) (Supplementary Figure S4). *Euryarchaeota* organisms also showed the dominance in the sediment samples from Dianchi Lake (56.6–92.3%) and Erhai Lake (48.8–76.2%). In either spring or summer, the proportion of sediment *Euryarchaeota* organisms illustrated a remarkable spatial change in these two freshwater lakes. Moreover, at a given sampling site in Dianchi Lake, the summer sediment sample generally showed lower *Euryarchaeota* proportion than the corresponding spring one. An opposite trend was observed in Erhai Lake. Phylum *Bathyarchaeota* also comprised a considerable proportion in the sediment samples from Dianchi Lake (2.2–36.6%) and Erhai Lake (18.6–36.2%). In either spring or summer, the proportion of sediment *Bathyarchaeota* organisms illustrated a remarkable spatial variation in these two freshwater lakes. At a given sampling site in Dianchi Lake, the sediment *Bathyarchaeota* proportion generally increased in summer, but an opposite trend occurred in Erhai Lake. Compared to *Bathyarchaeota*, *Lokiarchaeota* showed lower proportion in sediments of both Dianchi Lake (2.5–14.8%) and Erhai Lake (0.9–5.8%). In addition, *Thaumarchaeota* and *Crenarchaeota* were the minor archaeal groups in in sediments of both Dianchi Lake (*Thaumarchaeota* 0.1–0.6%, *Crenarchaeota* 0.4–1.5%) and Erhai Lake (*Thaumarchaeota* 0.6–8.6%, *Crenarchaeota* 0.5–3.3%).

In this study, a total of 13 archaeal classes were identified from Dianchi Lake and Erhai Lake, including *Thermoplasmata*, *Halobacteria*, *Methanomicrobia*, *Methanobacteria*, *Thermoprotei*, *Miscellaneous_Crenarchaeotic _Group*, *Marine_Benthic_Group_A*, *Marine_Benthic_Group_B*, *Marine_Group_I*, *Group_C3*, *AK8*, *AK59* and *pSL12*. *Marine_Benthic_Group_A* and *AK8* were usually classified as *Crenarchaeota* (Dang et al., 2010; Bougouffa et al., 2013; Hugoni et al., 2015), however, they were re-assigned as *Thaumarchaeota* in the Silva database. Phylogenetic analysis also indicated that they were more closely related to other known *Thaumarchaeota* classes (*AK59*, *Marine_Group_I* and *pSL12*) than to *Thermoprotei* (a *Crenarchaeota* class) (Supplementary Figure S5).

**FIGURE 5:**
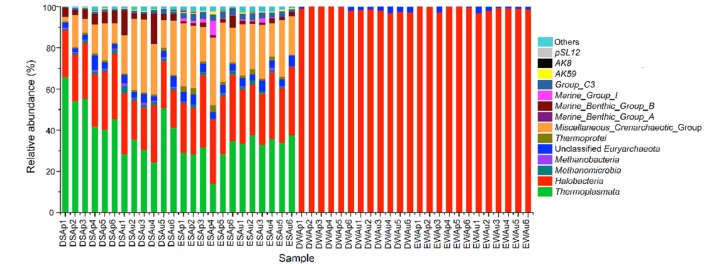
**Comparison of the quantitative contribution of the sequences affiliated with different archaeal classes to the total number of sequences from a given lake water or sediment sample.** The archaeal sequences that could not be affiliated with known phylum are included as “others”. Samples DWAp1, DWAp2, DWAp3, DWAp4, DWAp5, and DWAp6 represent the April water samples from sites D1–D6 in Dianchi Lake, respectively. Samples DWAu1, DWAu2, DWAu3, DWAu4, DWAu5, and DWAu6 represent the August water samples from sites D1–D6 in Dianchi Lake, respectively. Samples EWAp1, EWAp2, EWAp3, EWAp4, EWAp5, and EWAp6 represent the April water samples from sites E1–E6 in Erhai Lake, respectively. Samples EWAu1, EWAu2, EWAu3, EWAu4, EWAu5, and EWAu6 represent the August water samples from sites E1–E6 in Erhai Lake, respectively. Samples DSAp1, DSAp2, DSAp3, DSAp4, DSAp5, and DSAp6 represent the April sediment samples from sites D1–D6 in Dianchi Lake, respectively. Samples DSAu1, DSAu2, DSAu3, DSAu4, DSAu5, and DSAu6 represent the August sediment samples from sites D1–D6 in Dianchi Lake, respectively. Samples ESAp1, ESAp2, ESAp3, ESAp4, ESAp5, and ESAp6 represent the April sediment samples from sites E1–E6 in Erhai Lake, respectively. Samples ESAu1, ESAu2, ESAu3, ESAu4, ESAu5, and ESAu6 represent the August sediment samples from sites E1–E6 in Erhai Lake, respectively.

*Halobacteria* (within *Euryarchaeota*) was the predominant archaeal class in lake waters (accounting for ≥96.7%) (**Figure 5**). *Halobacteria* organisms was one of the major class groups in sediments of both Dianchi Lake (18.4–31.8%) and Erhai Lake (22.6–35%). In either spring or summer, the proportion of sediment *Halobacteria* organisms displayed a considerable spatial variation in these two lakes. At most of sampling sites in Dianchi Lake (4 out of 6), the sediment *Halobacteria* proportion decreased in summer, while an opposite trend was found in Erhai Lake. Moreover, *Thermoplasmata* (within *Euryarchaeota*) was also a major archaeal class in sediments of both Dianchi Lake (24.4–65.8%) and Erhai Lake (14–37.5%). The *Thermoplasmata* proportion showed a remarkable spatial variation in both spring and summer sediments of Dianchi Lake and in spring sediments of Erhai Lake, but a slight one in summer sediments of Erhai Lake. At a given sampling site in Dianchi Lake, the sediment *Thermoplasmata* proportion generally decreased in summer. In contrast, the sediment *Thermoplasmata* proportion showed an increase in summer at each sampling site in Erhai Lake. In addition, *Miscellaneous_Crenarchaeotic_Group* (within *Bathyarchaeota*) showed a relatively high proportion in sediments of both Dianchi Lake (1.9–35.5%) and Erhai Lake (16.1–32.9%). In either spring or summer, the proportion of *Miscellaneous_Crenarchaeotic_Group* demonstrated a large spatial variation in both Dianchi Lake and Erhai Lake. At most of sampling sites in Dianchi Lake (5 out of 6), the proportion of sediment *Miscellaneous_Crenarchaeotic_Group* increased in summer. However, the proportion of sediment *Miscellaneous_Crenarchaeotic_Group* illustrated a decrease in summer at most of sampling sites in Erhai Lake (4 out of 6).

### Influential Factors Regulating Archaeal Community

**FIGURE 6:**
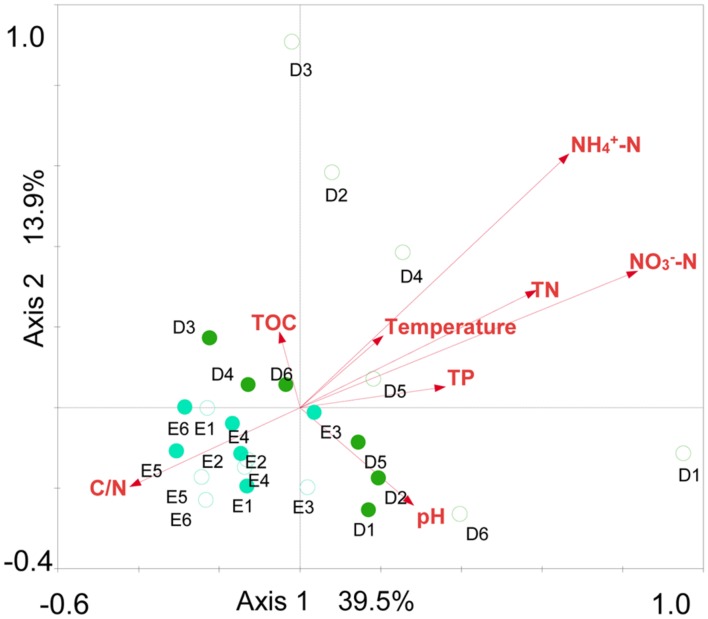
**Redundancy analysis (RDA) ordination plot for the first two principal dimensions of the relationships between archaeaplankton OTU composition and lake water parameters.** Green filled and open circles indicate the spring and summer water samples at sites D1–D6 in Dianchi Lake, respectively. Blue filled and open circles indicate the spring and summer water samples at sites E1–E6 in Erhai Lake, respectively.

Spearman rank correlation analysis indicated that archaeaplankton abundance was negatively correlated to the level of total organic carbon (TOC) in lake water (*P* < 0.01) (**Table 1**). Archaeaplankton Chao1 richness illustrated significant positive correlations with the levels of ammonia nitrogen, nitrate nitrogen, total nitrogen (TN), and total phosphorous (TP) (*P* < 0.05 or *P* < 0.01), but a negative correlation with the ratio of TOC to TN (C/N) (*P* < 0.05). Water temperature and nitrate nitrogen were positively correlated to archaeaplankton Shannon diversity (*P* < 0.01), but negatively to the *Euryarchaeota* and *Halobacteria* proportion (*P* < 0.05 or *P* < 0.01). The water environmental factors in the first two RDA axes respectively accounted for 39.5% and 13.9 % of the total variance for archaeaplankton OTU composition (**Figure 6**). In the current study, NO_3_^-^-N (*F* = 3.57 *P* = 0.002, 499 permutations), NH_4_^+^-N (*F* = 2.80, *P* = 0.002, 499 permutations), and TN (*F* = 2.40, *P* = 0.006, 499 permutations) significantly contributed to the archaeaplankton–environment relationship.

**Table 1:** Spearman rank correlation analysis of water environmental factors with the abundance, richness, and diversity of archaeaplankton community or the proportion of the major planktonic archaeal groups.

	pH	Temperature	NH_4_^+^-N	NO_3_^-^-N	TN	TP	TOC	C/N
Abundance	0.036	0.271	-0.259	0.293	-0.143	-0.018	-0.612ˆ**	-0.332
Operational taxonomic units (OTUs)	-0.394	-0.082	0.072	-0.009	0.005	-0.084	-0.043	-0.01
Chao1 richness	-0.003	0.117	0.651ˆ**	0.737ˆ**	0.624ˆ**	0.481ˆ*	0.227	-0.438ˆ*
Shannon diversity	-0.178	0.681ˆ**	0.308	0.568ˆ**	0.061	0.146	-0.28	-0.326
*Euryarchaeota*	-0.018	-0.427ˆ*	-0.23	-0.484ˆ*	-0.1	0.08	0.17	0.33
*Halobacteria*	0.31	-0.575ˆ**	-0.188	-0.424ˆ*	0.049	0.084	0.19	0.21

*^∗^Correlation is significant at the 0.05 level; ^∗∗^Correlation is significant at the 0.01 level. TN, total nitrogen; TP, total phosphorous; TOC, total organic carbon; C/N, the ratio of TOC to TN.*

**FIGURE 7:**
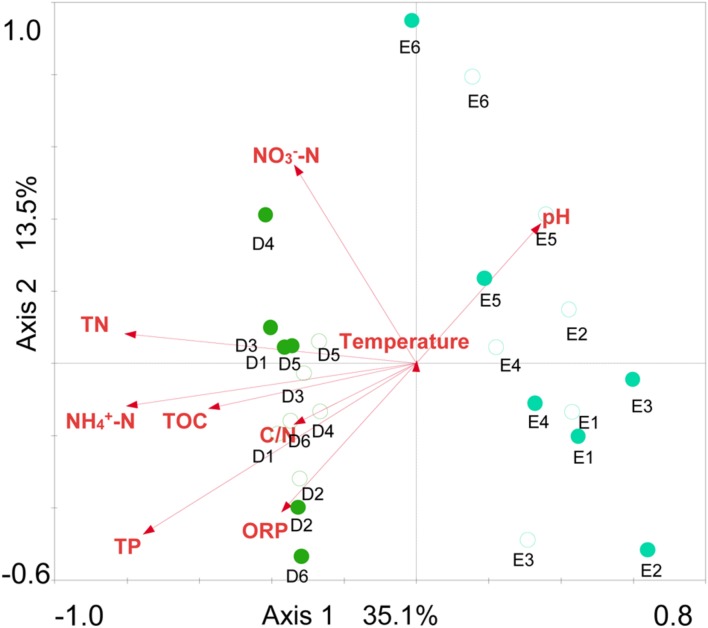
**Redundancy analysis ordination plot for the first two principal dimensions of the relationships between sediment archaeal OTU composition and sediment parameters.** Green filled and open circles indicate the spring and summer sediment samples at sites D1–D6 in Dianchi Lake, respectively. Blue filled and open circles indicate the spring and summer sediment samples at sites E1–E6 in Erhai Lake, respectively.

Sediment archaeal abundance was negatively correlated to sediment temperature and C/N (*P* < 0.01), but positively to NH_4_^+^-N, TN and TP (*P* < 0.01) (**Table 2**). Sediment archaeal OTUs, Chao1 richness and Shannon diversity showed positive correlations with pH (*P* < 0.05 or *P* < 0.01), but negative correlations with oxidation and reduction potential (ORP), NH_4_^+^-N, NO_3_^-^-N, TN, TP, and TOC (*P* < 0.05 or *P* < 0.01). Sediment *Euryarchaeota* proportion was positively correlated to NH_4_^+^-N and TN (*P* < 0.05). NH_4_^+^-N, NO_3_^-^-N, and TN were positively correlated to sediment *Thermoplasmata* proportion (*P* < 0.05 or *P* < 0.01), but negatively to sediment *Bathyarchaeota* or *Miscellaneous_Crenarchaeotic_Group* proportion (*P* < 0.05 or *P* < 0.01). Sediment *Halobacteria* proportion displayed negative correlations with ORP and TP (*P* < 0.05). The sediment environmental factors in the first two RDA axes, respectively, explained 35.1 and 13.5% of the total variance for sediment archaeal OTU composition (**Figure 7**). Sediment environmental factors including TN (*F* = 3.16; *P* = 0.002, 499 permutations), NH_4_^+^-N (*F* = 3.10; *P* = 0.002, 499 permutations), TP (*F* = 2.99; *P* = 0.002, 499 permutations), and TOC (*F* = 1.83, *P* = 0.014, 499 permutations) displayed significant contribution to the sediment archaeal population–environment relationship.

**Table 2:** Spearman rank correlation analysis of sediment environmental factors with the abundance, richness, and diversity of sediment archaeal community or the proportion of the major sediment archaeal groups.

	pH	Temperature	ORP	NH_4_^+^-N	NO_3_^-^-N	TN	TP	TOC	C/N
Abundance	-0.045	-0.779ˆ**	-0.137	0.548ˆ**	0.227	0.79ˆ**	0.554ˆ**	0.028	-0.541ˆ**
OTUs	0.449ˆ*	0.097	-0.445ˆ*	-0.758ˆ**	-0.427ˆ*	-0.708ˆ**	-0.767ˆ**	-0.626ˆ**	-0.203
Chao1 richness	0.526ˆ**	0.021	-0.577ˆ**	-0.632ˆ**	-0.452ˆ*	-0.623ˆ**	-0.745ˆ**	-0.501ˆ*	-0.199
Shannon diversity	0.424ˆ*	0.004	-0.437ˆ*	-0.734ˆ**	-0.583ˆ**	-0.699ˆ**	-0.677ˆ**	-0.575ˆ**	-0.195
*Euryarchaeota*	-0.254	-0.215	0.05	0.408ˆ*	0.372	0.494ˆ*	0.205	0.045	-0.268
*Bathyarchaeota*	0.379	0.238	-0.143	-0.501ˆ*	-0.476ˆ*	-0.570ˆ**	-0.273	-0.125	0.205
*Thermoplasmata*	-0.346	-0.075	0.173	0.428ˆ*	0.443ˆ*	0.541ˆ**	0.375	0.104	-0.203
*Halobacteria*	0.34	-0.188	-0.449ˆ*	-0.224	-0.028	-0.117	-0.405ˆ*	-0.167	-0.204
*Miscellaneous_Crenarchaeotic_Group*	0.327	0.265	-0.098	-0.456ˆ*	-0.419ˆ*	-0.496ˆ*	-0.201	-0.077	0.211

*^∗^Correlation is significant at the 0.05 level; ^∗∗^Correlation is significant at the 0.01 level. ORP, oxidation and reduction potential; TN, total nitrogen; TP, total phosphorous; TOC, total organic carbon; C/N, the ratio of TOC to TN.*

## Discussion

### Archaeal Abundance in Freshwater Lake

Information on planktonic archaeal abundance in freshwater lake is still limited. Several previous studies have revealed the seasonal or temporal change of archaeaplankton abundance in freshwater lake (Pernthaler et al., 1998; Callieri et al., 2009; Vila-Costa et al., 2013), while only an earlier report illustrated the horizontal variation of archaeaplankton abundance in freshwater lake (Keough et al., 2003). Moreover, little is known about the difference in archaeaplankton abundance among different freshwater lakes (Auguet and Casamayor, 2008). The obtained archaeaplankton abundance in these previous studies were based on 4,6-diamidino-2-phenylindole (DAPI) counts and fluorescence *in situ* hybridization (FISH) counts. So far, the links between environmental factors and archaeaplankton abundance in freshwater lake remain unclear. To the authors’ knowledge, this was the first study to systematically compare the dynamics of archaeaplankton abundance with sampling site and time in freshwater lakes at different trophic status. In the present study, qPCR targeting archaeal 16S rRNA gene was used to quantify archaeaplankton abundance in eutrophic Dianchi Lake and mesotrophic Erhai Lake. Archaeaplankton abundance in Dianchi Lake and Erhai Lake was 1.03 ± 0.07 × 10^7^– 8.2 ± 0.48 × 10^7^ and 6.75 ± 1.71 × 10^5^ – 1.75 ± 0.06 × 10^8^ 16S rRNA gene copies per L water, respectively. Planktonic archaeal community had much lower abundance than planktonic bacterial community in Dianchi Lake, while their abundance were comparable in Erhai Lake (Dai et al., 2015). In either Dianchi Lake or Erhai Lake, archaeaplankton abundance in summer was evidently different from that in spring. In summer, archaeaplankton abundance considerably decreased in Dianchi Lake but increased in Erhai Lake. Dianchi Lake showed higher archaeaplankton abundance than Erhai Lake in spring, but an opposite trend was observed in summer. Hence, the present study provided the evidence that the difference of freshwater lake archaeaplankton abundance between in spring and summer was lake-specific. In contrast, Dianchi Lake showed higher planktonic bacterial abundance than Erhai Lake in both spring and summer (Dai et al., 2015). In addition, a considerable spatial change of archaeaplankton abundance occurred in Dianchi Lake. This was in agreement with the results reported in the previous studies (Pernthaler et al., 1998; Callieri et al., 2009; Vila-Costa et al., 2013). However, in Erhai Lake, archaeaplankton abundance illustrated a remarkable spatial variation in summer but only a slight one in spring. This suggested that the spatial change of archaeaplankton abundance might be lake- and time-specific. The result of Spearman rank correlation analysis suggested that water TOC might be a key determinant of archaeaplankton abundance in Dianchi Lake and Erhai Lake. To the authors’ knowledge, this was the first report on the potential influence of TOC on archaeaplankton abundance in freshwater lake. Our previous study suggested that lake trophic status might be a key determinant to bacterioplankton abundance in these two lakes (Dai et al., 2015). Hence, the variables regulating archaeaplankton abundance might be different from that regulating bacterioplankton abundance in freshwater lake.

There have been only several reports on sediment archaeal abundance in freshwater lake ecosystems. Schwarz et al. (2007) revealed a slight temporal fluctuation of archaeal abundance in profundal sediment in Lake Kinneret. Two previous studies displayed the sediment depth-related change of archaeal abundance in Lake Pavin (Borrel et al., 2012) and LakeTaihu (Ye et al., 2009). Our recent study reported the difference of archaeal abundance in profundal sediments of small freshwater lakes on the Yunnan Plateau (Zhang et al., 2015). All of these previous studies applied qPCR to quantify lake sediment archaeal abundance. To date, information on the horizontal change of sediment archaeal abundance in a given freshwater lake is still lacking. The environmental variables driving the shift in sediment archaeal abundance remain unknown. To the authors’ knowledge, this was also the first study to systematically compare the dynamics of sediment archaeal abundance with sampling site and time in different freshwater lakes. In this study, qPCR was used to quantify sediment archaeal abundance in Dianchi Lake and Erhai Lake. Sediment archaeal abundance in Dianchi Lake and Erhai Lake was 1.4 ± 0.2 × 10^9^–1.45 ± 0.02 × 10^10^ and 3.3 ± 0.65 × 10^8^–4.95 ± 0.11 × 10^9^ 16S rRNA gene copies per gram dry sediment, respectively, higher than that in meso-eutrophic Lake Kinneret and oligomesotrophic Lake Pavin (Schwarz et al., 2007; Borrel et al., 2012) but lower than that in eutrophic Lake Taihu (Ye et al., 2009). In either spring or summer, eutrophic Dianchi Lake tended to have higher sediment archaeal abundance than mesotrophic Erhai Lake. Sediment archaeal community had much higher abundance than sediment bacterial community in Dianchi Lake, but their abundance was comparable in Erhai Lake (Dai et al., 2015). The result of Spearman rank correlation analysis also suggested that sediment archaeal abundance was likely influenced by the variables associated with the trophic status including NH_4_^+^-N, TN, and TP. Therefore, sediment archaeal abundance in freshwater lake might be determined by trophic status. Our previous study indicated that sediment bacterial abundance in Dianchi Lake and Erhai Lake might be influenced by pH, ORP, TOC, and C/N (Dai et al., 2015). Hence, the variables regulating sediment archaeal abundance might be different from that regulating bacterial archaeal abundance in freshwater lake. Moreover, in summer, sediment archaeal abundance considerably decreased in both Dianchi Lake and Erhai Lake. The result of Spearman rank correlation analysis further confirmed the negative effect of temperature rise on sediment archaeal abundance. In addition, in both Dianchi Lake and Erhai Lake, sediment archaeal abundance showed a remarkable spatial change in spring but a slight one in summer. This suggested that the spatial change of sediment archaeal abundance in freshwater lake might be time-specific.

### Archaeal Community Richness and Diversity in Freshwater Lake

The archaeaplankton community richness and diversity in freshwater lake has been usually investigated using traditional low-profiling molecular biology tools, such as terminal restriction fragment length polymorphism (TRFLP; Lehours et al., 2005), denaturing gradient gel electrophoresis (DGGE; Casamayor et al., 2000, 2001), and clone library (Lehours et al., 2007; Berdjeb et al., 2013). The application of high-throughput sequencing analysis to depict freshwater lake archaeaplankton community richness and diversity is still very limited (Vila-Costa et al., 2013; Li et al., 2015). Several previous studies suggested the marked seasonal or temporal change of archaeaplankton richness and diversity in freshwater lake (Casamayor et al., 2001; Berdjeb et al., 2013; Vila-Costa et al., 2013; Li et al., 2015), whereas Casamayor et al. (2000) indicated that the seasonal change of lake archaeaplankton DGGE pattern was not evident. Moreover, few previous studies compared the difference of archaeaplankton richness and diversity in various freshwater lake systems (Casamayor et al., 2001; Berdjeb et al., 2013; Vila-Costa et al., 2013). To date, there is a paucity of knowledge on the environmental factors driving the dynamics of archaeaplankton richness and diversity in freshwater lake. In this study, Illumina MiSeq high-throughput sequencing was used to monitor the dynamics of archaeaplankton communities in Dianchi Lake and Erhai Lake. The planktonic archaeal OTU number, Chao1 richness and Shannon diversity index in Dianchi Lake and Erhai Lake were 44–262 and 44–360, 64–331 and 42–132, and 1.6–3.32 and 1.69–2.16, respectively. The observed archaeal OTU number was comparable to that reported in other freshwater lakes using 454 pyrosequencing analysis (Vila-Costa et al., 2013). Archaeaplankton community in either Dianchi Lake or Erhai Lake had much lower OTU number, Chao1 richness and Shannon diversity index than bacterioplankton community (Dai et al., 2015). The evident spatial change of archaeaplankton richness was found in both Dianchi Lake and Erhai Lake. The variation of archaeaplankton richness in two different seasons could be observed at a given site in these two freshwater lakes, which was consistent with the results reported in other freshwater lakes (Casamayor et al., 2001; Berdjeb et al., 2013; Vila-Costa et al., 2013; Li et al., 2015). In addition, Dianchi Lake tended to have higher archaeaplankton richness than Erhai Lake. Spearman rank correlation analysis indicated that archaeaplankton richness showed positive correlations with the variables associated with the trophic status including NH_4_^+^-N, NO_3_^-^-N, TN, and TP (*P* < 0.05). Therefore, archaeaplankton richness in freshwater lake might be determined by trophic status. A remarkable spatial variation of archaeaplankton diversity occurred in Dianchi Lake, but only a slight one in Erhai Lake. This suggested that the spatial variation of archaeaplankton diversity might be lake-specific. In either of the two lakes, archaeaplankton diversity increased in summer. The result of Spearman rank correlation analysis further confirmed the positive influence of temperature rise on archaeaplankton diversity. Spearman rank correlation analysis suggested that nitrate nitrogen might also play an important role in determining archaeaplankton diversity.

Our previous study indicated that trophic status might be also an important driving force for bacterioplankton community richness and diversity in Dianchi Lake and Erhai Lake (Dai et al., 2015).

A few previous studies documented the evident horizontal change of sediment archaeal richness in freshwater lake ecosystem (Liu et al., 2009; Haller et al., 2011; Bhattarai et al., 2012), yet direct information is missing about the horizontal change of sediment archaeal diversity. The seasonal effect on sediment archaeal richness and diversity remains unclear. A slight seasonal shift in sediment archaeal richness and diversity occurred in Lake Kinneret (Schwarz et al., 2007), whereas a profound seasonal effect was observed in a Cerrado lake (Rodrigues et al., 2014). All of these previous studies were based on traditional low-profiling molecular biology tools. High-throughput sequencing has found a number of applications in depicting sediment archaeal community (Xie et al., 2014; Hu et al., 2015; Liu et al., 2015; Polymenakou et al., 2015), yet information about its application in freshwater lake sediment archaeal community is still very limited. Our recent study applied Illumina MiSeq high-throughput sequencing to compare the difference of archaeal community richness and diversity in profundal sediments of various freshwater lakes (Zhang et al., 2015). To date, the environmental factors regulating sediment archaeal richness and diversity in freshwater lake remain elusive. In this study, Illumina MiSeq sequencing was applied to monitor the dynamics of sediment archaeal communities in Dianchi Lake and Erhai Lake. The sediment archaeal OTU number, Chao1 richness and Shannon diversity index in Dianchi Lake and Erhai Lake were 543–782 and 950–1135, 858–1136 and 1210–1572, and 2.99–4.3 and 4.46–5.23, respectively, generally much lower than the reported values for profundal sediments in small freshwater lakes on the Yunnan Plateau (Zhang et al., 2015). Sediment archaeal community in either Dianchi Lake or Erhai Lake had much lower OTU number, Chao1 richness and Shannon diversity index than sediment bacterial community (Dai et al., 2015). The result of Spearman rank correlation analysis revealed that sediment archaeal OTU number, Chao1 richness and Shannon diversity were negatively correlated with a number of variables associated with the trophic status including NH_4_^+^-N, NO_3_^-^-N, TN, and TP (*P* < 0.05). To the authors’ knowledge, the present report provided the evidence for the first time that lake trophic status played a crucial role in determining sediment archaeal richness and diversity. Our previous study also suggested that sediment bacterial richness and diversity in Dianchi Lake and Erhai Lake might be influenced by lake trophic status (Dai et al., 2015). Eutrophic Dianchi Lake had much lower sediment archaeal richness and diversity than mesotrophic Erhai Lake. Dianchi Lake also had lower sediment bacterial richness and diversity than Erhai Lake (Dai et al., 2015). Sediment archaeal richness and diversity might be also influences by other environmental factors (pH, ORP and TOC). The evident spatial change of sediment archaeal richness was found in both Dianchi Lake and Erhai Lake. This was in consistency with the previous studies (Liu et al., 2009; Haller et al., 2011; Bhattarai et al., 2012). The present study further displayed the evident spatial variation of sediment archaeal diversity. The difference of sediment archaeal richness between in spring and summer could be usually observed at a given site in these two freshwater lakes. This was in agreement with the result observed for a Cerrado lake (Rodrigues et al., 2014). Sediment archaeal diversity in Erhai Lake slightly increased in summer, whereas no clear trend of diversity shift was observed in Dianchi Lake. This suggested that the change of sediment archaeal diversity with sampling time might be lake-specific. To date, no information exists on the discrepancy of archaeal richness or diversity between in water column and in sediment of freshwater lake. The current study revealed that sediments had much higher archaeal richness and diversity than waters. In both Dianchi Lake and Erhai Lake, bacterial richness and diversity were also higher in sediments than in waters (Dai et al., 2015).

### Archaeal Community Structure in Freshwater Lake

The seasonal or temporal change of archaeaplankton community structure in freshwater lake has been well-documented (Casamayor et al., 2001; Lliros et al., 2008; Berdjeb et al., 2013; Vila-Costa et al., 2013; Li et al., 2015). In the present study, the results of UPGMA clustering indicated an apparent difference of archaeaplankton community structure between in spring and summer in either Dianchi Lake or Erhai Lake. Our previous study also revealed the temporal change of bacterioplankton community structure in either of these two lakes (Dai et al., 2015). A remarkable difference of bacterioplankton community structure occurred between in Dianchi Lake and Erhai Lake (Dai et al., 2015). In this study, Dianchi Lake and Erhai Lake also differed in archaeaplankton community structure. Some previous studies also illustrated the evident discrepancy of archaeaplankton community structure between (or among) different freshwater lakes (Casamayor et al., 2000, 2001; Auguet and Casamayor, 2008; Vila-Costa et al., 2013). Hence, archaeaplankton community structure might be lake-specific. Moreover, in either Dianchi Lake or Erhai Lake, the result of UPGMA clustering suggested that the temporal variation of archaeaplankton community was greater than the spatial variation. This was consistent with the result reported for Lake Taihu (Li et al., 2015). So far, the links between environmental variables and archaeaplankton community structure remain not well understood. Berdjeb et al. (2013) suggested that archaeaplankton community structure might be regulated by a number of environmental factors such as temperature, nutrients, chlorophyll a and dissolved oxygen. In this study, the result of RDA suggested that trophic status might play a crucial role in shaping archaeaplankton community structure. Nutrients as well as temperature might shape bacterioplankton community structure in Dianchi Lake and Erhai Lake (Dai et al., 2015). The slight temporal shift in sediment archaeal community structure has been reported in Lake Kinneret (Schwarz et al., 2007) and Lake Taihu (Chen et al., 2015). In contrast, Rodrigues et al. (2014) showed the considerable seasonality of sediment archaeal community structure in a Cerrado lake. In this study, the result of UPGMA clustering suggested the slight difference of sediment archaeal community structure between in spring and summer in Erhai Lake, but the remarkable difference in Dianchi Lake. Therefore, the difference of sediment archaeal community structure between in spring and summer might be lake-specific. Our previous studies illustrated that sediment bacterial community structure in Dianchi Lake was distinctly different from that in Erhai Lake (Dai et al., 2015). In this study, the distinct structure difference of sediment archaeal communities was found between in Dianchi Lake and in Erhai Lake. Small freshwater lakes on the Yunnan Plateau also showed the evident discrepancy of sediment archaeal community structure (Zhang et al., 2015). These two studies suggested that sediment archaeal community structure might be also lake-specific. In addition, the present study provided the evidence for the first time that lake water and sediment had distinct archaeal community structures. The environmental factors driving the spatiotemporal dynamics of lake sediment archaeal community remain poorly documented. Chen et al. (2015) suggested the potential role of trophic level in shaping lake sediment archaeal community. In this study, the result of RDA suggested that TN, NH_4_^+^-N, TP, and TOC might collectively shape lake sediment archaeal community structure. These environmental factors also collectively structure sediment bacterial community in Dianchi Lake and Erhai Lake (Dai et al., 2015).

Phylogenetic analysis of archaeal communities indicated that phylum *Euryarchaeota* predominated in waters of both Dianchi Lake and Erhai Lake (accounting for ≥99.9%), but showed relatively lower proportion in sediments of Dianchi Lake (56.6–92.3%) and Erhai Lake (48.8–76.2%). Phylum *Bathyarchaeota* also accounted for a considerable proportion in sediments of both Dianchi Lake (2.2–36.6%) and Erhai Lake (18.6–36.2%). At class level, *Halobacteria* was the predominant archaeal class in lake waters (accounting for ≥96.7%), while sediment archaeal communities in Dianchi Lake and Erhai Lake were mainly composed of *Halobacteri*a (18.4–31.8% or 22.6–35%), *Thermoplasmata* (24.4–65.8% or 14–37.5%) and *Miscellaneous_Crenarchaeotic_Group* (1.9–35.5% or 16.1–32.9%). The Venn diagrams also demonstrate that, in either Dianchi Lake or Erhai Lake only a small proportion of OTUs detected in sediment was identified in water. These results further confirmed that water and sediment habitats differed greatly in archaeal community structure. To date, there has been no consensus on the dominant archaeaplankton phylum in freshwater lake. Several previous studies displayed the dominance of *Euryarchaeota* in freshwater lake archaeaplankton community (Lehours et al., 2007; Auguet and Casamayor, 2008; Vila-Costa et al., 2013). This was consistent with the current study. In contrast, Li et al. (2015) suggested that *Crenarchaeota* organisms were more abundant than *Euryarchaeota* organisms in Lake Taihu. Casamayor et al. (2000) found that *Euryarchaeota* dominated in one lake, but *Crenarchaeota* in another lake. Berdjeb et al. (2013) even suggested the predominance of phylum *Thaumarchaeota* in two deep freshwater lakes. So far, the links between *Euryarchaeota* and environmental factors in freshwater remain unclear. In this study, the result of Spearman rank correlation analysis suggested that the *Euryarchaeota* proportion in archaeaplankton community might be negatively influenced by water temperature and nitrate level.

*Euryarchaeota* organisms were usually found to predominate in freshwater lake sediment archaeal community (Schwarz et al., 2007; Liu et al., 2009; Ye et al., 2009; Haller et al., 2011; Bhattarai et al., 2012; Borrel et al., 2012; Kadnikov et al., 2012). In this study, *Euryarchaeota* organisms showed the dominance in sediments of both Dianchi Lake and Erhai Lake, but *Bathyarchaeota* organisms also showed a considerable proportion. The present study illustrated a remarkable spatial fluctuation of the sediment *Euryarchaeota* and *Bathyarchaeota* proportion in both Dianchi Lake and Erhai Lake, further indicating the evident spatial change of sediment archaeal community structure. In Dianchi Lake, in summer, the sediment *Bathyarchaeota* proportion generally increased, while the *Euryarchaeota* proportion generally decreased. An opposite trend was observed in Erhai Lake. These results suggested that the temporal change of sediment *Euryarchaeota and Bathyarchaeota* proportion were lake-specific. To date, the environmental factors influencing the *Euryarchaeota* and *Bathyarchaeota* proportion in freshwater lake sediment remain unknown. In this study, the result of Spearman rank correlation analysis suggested that the *Euryarchaeota* proportion was positively influenced by the levels of sediment ammonia nitrogen and TN, while the *Bathyarchaeota* proportion was negatively influenced by ammonia nitrogen, nitrate nitrogen and TN. Hence, sediment nitrogen level might be a key determinant of sediment archaeal community structure in freshwater lake, which was also sustained by the result of RDA. Chen et al. (2015) suggested that archaeal community structure was sensitive to the sediment trophic level in Lake Taihu.

## Conclusion

Dianchi Lake and Erhai Lake had distinct both planktonic and sediment archaeal communities, which might be determined by lake trophic status. Water and sediment habitats also differed greatly in archaeal community structure. *Euryarchaeota* showed the dominance in waters and sediments of both Dianchi Lake and Erhai Lake, but *Bathyarchaeota* was also an important component in lake sediments.

## Author Contributions

Conceived and designed the experiments: SX and YL; Performed the experiments: YY, YD, and ZW; Analyzed the data: YY and YD; Wrote the paper: SX.

## Conflict of Interest Statement

The authors declare that the research was conducted in the absence of any commercial or financial relationships that could be construed as a potential conflict of interest.
