# Supplementary material for: Temporal and Spatial Dynamics of Archaeal Communities in Two Freshwater Lakes at Different Trophic Status
Source: Front Microbiol. 2016 Mar 31;7:451. doi: 10.3389/fmicb.2016.00451 (PMC4814500; doi:10.3389/fmicb.2016.00451)

**Supplementary Material**

**Journal name:** ***Frontiers in Microbiology***

**Manuscript title:** Temporal and spatial dynamics of archaeal communities in two freshwater lakes at different trophic status

Yuyin Yang^a,#^, Yu Dai^a,#^, Zhen Wu^b^, Shuguang Xie^a,*^, Yong Liu^b,*^

^#^ Yuyin Yang and Yu Dai contributed equally to this study.

^a^State Key Joint Laboratory of Environmental Simulation and Pollution Control, College of Environmental Sciences and Engineering, Peking University, Beijing 100871, China

^b^Key Laboratory of Water and Sediment Sciences (Ministry of Education), College of Environmental Sciences and Engineering, Peking University, Beijing 100871, China

* Corresponding author. Tel: 86-10-62751923. Fax: 86-10-62751923.

Email: [xiesg@pku.edu.cn](mailto:xiesg@pku.edu.cn) (S Xie); yongliu@pku.edu.cn (Y Liu)

**Table S1** Percentage of the sequences belonging to major OTUs (with relative abundance of no less 1% in at least one sample) to the total number of sequences from a given water sample. Samples DWAp1, DWAp2, DWAp3, DWAp4, DWAp5 and DWAp6 represent the April water samples from sites D1–D6 in Dianchi Lake, respectively. Samples DWAu1, DWAu2, DWAu3, DWAu4, DWAu5 and DWAu6 represent the August water samples from sites D1–D6 in Dianchi Lake, respectively. Samples EWAp1, EWAp2, EWAp3, EWAp4, EWAp5 and EWAp6 represent the April water samples from sites E1–E6 in Erhai Lake, respectively. Samples EWAu1, EWAu2, EWAu3, EWAu4, EWAu5 and EWAu6 represent the August water samples from sites E1–E6 in Erhai Lake, respectively.

| OTU ID | DWAp1 | DWAp2 | DWAp3 | DWAp4 | DWAp5 | DWAp6 | DWAu1 | DWAu2 | DWAu3 | DWAu4 | DWAu5 | DWAu6 | EWAp1 | EWAp2 | EWAp3 | EWAp4 | EWAp5 | EWAp6 | EWAu1 | EWAu2 | EWAu3 | EWAu4 | EWAu5 | EWAu6 |
| --- | --- | --- | --- | --- | --- | --- | --- | --- | --- | --- | --- | --- | --- | --- | --- | --- | --- | --- | --- | --- | --- | --- | --- | --- |
| OTU893 | 48.55 | 59.02 | 57.34 | 56.2 | 54.37 | 48.16 | 10.78 | 16.81 | 12.24 | 13.5 | 12.21 | 11.03 | 45.74 | 41.35 | 49.22 | 29.16 | 28.35 | 23.21 | 1.67 | 0.73 | 2.1 | 3.62 | 1.66 | 1.29 |
| OTU210 | 5.17 | 2.03 | 2.74 | 2.07 | 2.19 | 2.43 | 2.14 | 3.92 | 6.11 | 5.58 | 8.11 | 9.68 | 7.62 | 10.47 | 16.89 | 17.28 | 24.83 | 37.23 | 31.61 | 39.75 | 43.06 | 40.71 | 40 | 40.91 |
| OTU1422 | 6.63 | 2.31 | 3.19 | 1.43 | 1.93 | 1.83 | 16.83 | 6.75 | 9.95 | 8.73 | 8.19 | 8.01 | 20.04 | 21.92 | 11.67 | 28.24 | 23.23 | 16.16 | 9.97 | 9.43 | 7.05 | 6.24 | 10.13 | 10.89 |
| OTU1582 | 12.4 | 15.7 | 13.54 | 15.41 | 17.93 | 15.69 | 8.86 | 32.57 | 19.57 | 23.17 | 25.27 | 19.74 | 1.32 | 1.44 | 2.34 | 1.7 | 2.49 | 2.74 | 0.13 | 0.24 | 0.14 | 0.44 | 0.46 | 0.54 |
| OTU2111 | 3.11 | 0.56 | 1.01 | 0.63 | 0.80 | 0.62 | 0.26 | 0.45 | 0.34 | 0.57 | 0.49 | 0.58 | 14.19 | 13.4 | 6.64 | 13.50 | 9.23 | 9.61 | 2.34 | 3.64 | 6.66 | 3.49 | 12.3 | 14.33 |
| OTU1473 | 9.02 | 7.71 | 7.49 | 11.92 | 8.07 | 7.51 | 1.69 | 3.89 | 5.94 | 5.23 | 5.06 | 4.87 | 0.18 | 0.25 | 1.33 | 0.44 | 0.92 | 1.01 | 2.15 | 2.11 | 3.91 | 3.68 | 5.8 | 5.11 |
| OTU352 | 0.16 | 0 | 0.02 | 0 | 0 | 0 | 0.15 | 0.22 | 0.23 | 0.24 | 0.18 | 0.25 | 0 | 0 | 0 | 0 | 0 | 0 | 28.88 | 21.7 | 12.04 | 7.36 | 7.25 | 6.93 |
| OTU2144 | 0.46 | 0.81 | 1.17 | 1.13 | 1.25 | 1.45 | 0.70 | 4.69 | 7.77 | 5.39 | 4.54 | 7.46 | 0.65 | 0.86 | 1.16 | 0.87 | 0.68 | 0.6 | 1.42 | 1.72 | 5.38 | 5.28 | 1.6 | 1.02 |
| OTU1423 | 0.23 | 0.4 | 0.28 | 0.52 | 0.36 | 0.33 | 4.16 | 9 | 8.27 | 12.24 | 10.5 | 10.6 | 0.03 | 0.04 | 0.12 | 0.08 | 0.1 | 0.13 | 0 | 0.03 | 0 | 0.01 | 0.08 | 0.01 |
| OTU2353 | 0.07 | 0.02 | 0.05 | 0.05 | 0.06 | 0.03 | 0.03 | 0.06 | 0.16 | 0.09 | 0.19 | 0.14 | 0.01 | 0.03 | 0.09 | 0.03 | 0.03 | 0.06 | 4.58 | 6.39 | 10.3 | 16.94 | 9.24 | 8.2 |
| OTU1471 | 0.06 | 0.02 | 0.02 | 0.02 | 0.07 | 0.05 | 6.00 | 4.63 | 7.16 | 6.44 | 6.42 | 6.63 | 0 | 0 | 0.01 | 0.00 | 0.00 | 0.00 | 4.84 | 3.17 | 1.73 | 1.47 | 1.9 | 1.77 |
| OTU855 | 0.68 | 0.9 | 1 | 1.03 | 1.25 | 1.51 | 0.71 | 2.40 | 3.55 | 3.05 | 3.19 | 3.95 | 2.17 | 2.26 | 1.51 | 0.98 | 1.38 | 1.44 | 0.7 | 1.09 | 1.43 | 2 | 1.14 | 0.72 |
| OTU448 | 1.61 | 0.23 | 0.5 | 0.33 | 0.42 | 0.41 | 2.18 | 1.74 | 2.2 | 3.08 | 2.47 | 2.41 | 1.43 | 1.8 | 1.36 | 2.18 | 2.84 | 2.56 | 0.55 | 1.30 | 0.25 | 0.47 | 1.45 | 2.01 |
| OTU121 | 2.08 | 1.24 | 1.19 | 1.17 | 1.32 | 1.33 | 1.04 | 0.83 | 0.68 | 0.75 | 0.61 | 0.44 | 3.98 | 3.59 | 2.23 | 2.95 | 2.22 | 1.53 | 0.02 | 0.06 | 0.01 | 0.01 | 0.15 | 0.14 |
| OTU224 | 0.12 | 0.03 | 0.15 | 0.02 | 0.07 | 0.04 | 9.34 | 3.24 | 3.59 | 1.50 | 2.81 | 2.37 | 0.23 | 0.23 | 0.1 | 0.1 | 0.1 | 0.08 | 0.13 | 0.23 | 0.03 | 0.02 | 0.17 | 0.12 |
| OTU1222 | 1.58 | 1.48 | 3.11 | 0.64 | 2.42 | 1.50 | 1.30 | 2.08 | 3.81 | 1.26 | 1.77 | 2.59 | 0 | 0 | 0 | 0 | 0 | 0 | 0.01 | 0 | 0.04 | 0 | 0 | 0 |
| OTU1106 | 0.87 | 0.38 | 0.51 | 0.3 | 0.41 | 0.38 | 0.54 | 0.28 | 0.22 | 0.31 | 0.31 | 0.36 | 0.1 | 0.16 | 0.36 | 0.44 | 1.28 | 1.78 | 2.23 | 1.85 | 2.47 | 3.23 | 1.54 | 1.24 |
| OTU1862 | 1 | 1.37 | 1.23 | 1.20 | 1.35 | 1.37 | 1.64 | 0.97 | 0.98 | 1.08 | 1.05 | 0.91 | 0.54 | 0.50 | 0.79 | 0.35 | 0.31 | 0.14 | 0.01 | 0.01 | 0 | 0.03 | 0.03 | 0.05 |
| OTU379 | 0 | 0 | 0 | 0.01 | 0 | 0 | 0.5 | 0.91 | 1.67 | 2.77 | 2.08 | 2.35 | 0 | 0 | 0 | 0 | 0 | 0 | 2.4 | 0.38 | 0.3 | 0.37 | 0.01 | 0.07 |
| OTU720 | 1.66 | 1.59 | 1.58 | 2.62 | 1.86 | 1.29 | 0.26 | 0.27 | 0.36 | 0.37 | 0.28 | 0.25 | 0.05 | 0.04 | 0.1 | 0.02 | 0.09 | 0.12 | 0.01 | 0.01 | 0.02 | 0.02 | 0.03 | 0.05 |
| OTU197 | 1.46 | 1.54 | 1.29 | 2.13 | 1.47 | 1.19 | 0.11 | 0.48 | 0.42 | 0.49 | 0.45 | 0.4 | 0.05 | 0.08 | 0.13 | 0.09 | 0.09 | 0.08 | 0.01 | 0.01 | 0.02 | 0.01 | 0.02 | 0.05 |
| OTU250 | 0.07 | 0 | 0.03 | 0.01 | 0.01 | 0 | 0.07 | 0.05 | 0.11 | 0.12 | 0.09 | 0.1 | 0.17 | 0.18 | 0.11 | 0.35 | 0.36 | 0.28 | 1.15 | 1.57 | 0.57 | 1.7 | 1.7 | 1.63 |
| OTU781 | 0.04 | 0.00 | 0.01 | 0 | 0 | 0 | 0.25 | 0.3 | 0.51 | 0.49 | 0.44 | 0.49 | 0 | 0 | 0 | 0 | 0 | 0 | 2.21 | 1.53 | 0.85 | 0.49 | 0.66 | 0.57 |
| OTU206 | 0.13 | 0.18 | 0.18 | 0.19 | 0.25 | 0.18 | 0.2 | 1.3 | 1.27 | 1.16 | 1.13 | 1.18 | 0.01 | 0.02 | 0.05 | 0.06 | 0.04 | 0.07 | 0.02 | 0.04 | 0.03 | 0.1 | 0.06 | 0.07 |
| OTU409 | 0.06 | 0.02 | 0.01 | 0.01 | 0.02 | 0.01 | 0.08 | 0.09 | 0.18 | 0.23 | 0.31 | 0.28 | 0.06 | 0.08 | 0.14 | 0.12 | 0.2 | 0.37 | 0.80 | 1.53 | 0.38 | 0.51 | 0.87 | 1.15 |
| OTU889 | 0 | 0 | 0 | 0 | 0 | 0.05 | 6.46 | 0.01 | 0 | 0 | 0.01 | 0.01 | 0 | 0 | 0 | 0 | 0 | 0 | 0 | 0 | 0 | 0 | 0 | 0 |

**Table S2** Percentage of the sequences belonging to major OTUs (with relative abundance of no less 1% in at least one sample) to the total number of sequences from a given sediment sample. Samples DSAp1, DSAp2, DSAp3, DSAp4, DSAp5 and DSAp6 represent the April sediment samples from sites D1–D6 in Dianchi Lake, respectively. Samples DSAu1, DSAu2, DSAu3, DSAu4, DSAu5 and DSAu6 represent the August sediment samples from sites D1–D6 in Dianchi Lake, respectively. Samples ESAp1, ESAp2, ESAp3, ESAp4, ESAp5 and ESAp6 represent the April sediment samples from sites E1–E6 in Erhai Lake, respectively. Samples ESAu1, ESAu2, ESAu3, ESAu4, ESAu5 and ESAu6 represent the August sediment samples from sites E1–E6 in Erhai Lake, respectively.

| OUT ID | DSAp1 | DSAp2 | DSAp3 | DSAp4 | DSAp5 | DSAp6 | DSAu1 | DSAu2 | DSAu3 | DSAu4 | DSAu5 | DSAu6 | ESAp1 | ESAp2 | ESAp3 | ESAp4 | ESAp5 | ESAp6 | ESAu1 | ESAu2 | ESAu3 | ESAu4 | ESAu5 | ESAu6 |
| --- | --- | --- | --- | --- | --- | --- | --- | --- | --- | --- | --- | --- | --- | --- | --- | --- | --- | --- | --- | --- | --- | --- | --- | --- |
| OTU2553 | 0.52 | 0.55 | 0.7 | 0.71 | 0.79 | 0.35 | 0.41 | 0.64 | 0.42 | 0.54 | 1.26 | 0.64 | 0.72 | 0.79 | 0.78 | 1.31 | 0.83 | 0.64 | 0.59 | 0.55 | 0.84 | 0.89 | 1.09 | 0.34 |
| OTU728 | 49.07 | 28.28 | 35.25 | 20.65 | 20.15 | 21.53 | 18.41 | 17.76 | 18.4 | 10.82 | 27.42 | 17.96 | 8.95 | 8.7 | 11.93 | 4.99 | 11.97 | 16.65 | 13.06 | 12.55 | 10.61 | 13.48 | 11.85 | 17.21 |
| OTU1880 | 1.11 | 0.94 | 0.91 | 3.98 | 5.76 | 12.36 | 2.7 | 1.44 | 4.32 | 8.15 | 2.15 | 1.45 | 1.5 | 1.84 | 8.14 | 1.77 | 3.97 | 3.15 | 3.91 | 3.19 | 3 | 5.71 | 2.7 | 2.04 |
| OTU40 | 2.11 | 5.77 | 2.37 | 3.15 | 3.93 | 3.89 | 0.78 | 3.24 | 1.55 | 1.87 | 3.96 | 8.87 | 1.36 | 1.24 | 2.17 | 0.5 | 1.63 | 2.67 | 2.18 | 2.64 | 1.26 | 2.27 | 1.82 | 2.22 |
| OTU1583 | 4.31 | 3.14 | 3.41 | 2.32 | 2.9 | 4.22 | 1.9 | 2.18 | 1.32 | 1.32 | 3.45 | 2.51 | 0.99 | 0.88 | 1.65 | 0.43 | 1.07 | 2.83 | 1.16 | 2.49 | 1.15 | 4.11 | 1.62 | 3.02 |
| OTU1582 | 4.9 | 3.86 | 5.23 | 0.75 | 1.53 | 0.53 | 0.51 | 2.21 | 0.61 | 0.44 | 0.49 | 1.22 | 0.65 | 0.72 | 2.4 | 0.79 | 1.33 | 4.7 | 0.65 | 0.67 | 0.69 | 2.14 | 2.18 | 10.73 |
| OTU463 | 1.55 | 2.63 | 2.08 | 1.66 | 1.35 | 1.47 | 0.75 | 1.93 | 1.06 | 0.96 | 2.53 | 2.96 | 1.01 | 1 | 0.95 | 0.49 | 1.02 | 0.91 | 1.82 | 1.55 | 1.1 | 1.25 | 0.99 | 1.28 |
| OTU1138 | 0.42 | 0.96 | 1.59 | 2.72 | 2.45 | 1.99 | 0.22 | 0.92 | 1.18 | 1.93 | 4.72 | 0.51 | 1.02 | 0.95 | 0.81 | 0.44 | 0.67 | 0.65 | 1.57 | 1.6 | 1.2 | 1.67 | 1.35 | 0.61 |
| OTU822 | 0.44 | 0.35 | 1.03 | 5.02 | 0.81 | 0.52 | 1.35 | 0.70 | 2.69 | 0.66 | 1.77 | 3.5 | 0.89 | 1.17 | 1.33 | 1.13 | 1.1 | 0.34 | 0.65 | 0.69 | 1.17 | 0.18 | 0.4 | 0.06 |
| OTU1440 | 1.78 | 4.32 | 3.2 | 2.03 | 1.99 | 2.62 | 1.03 | 2.24 | 1.18 | 1.3 | 1.51 | 1.97 | 0.02 | 0.02 | 0.06 | 0.02 | 0.09 | 0.05 | 0.21 | 0.03 | 0.06 | 0.1 | 0.09 | 0.01 |
| OTU1618 | 0.07 | 0.06 | 0.03 | 0.08 | 0.08 | 0.03 | 0.24 | 0.12 | 0.22 | 0.14 | 0.01 | 0.25 | 2.51 | 2.55 | 1.52 | 0.54 | 1.2 | 1.78 | 2.55 | 2.72 | 2.98 | 1.71 | 1.75 | 2.71 |
| OTU2201 | 0.64 | 0.85 | 3.27 | 2.78 | 2.22 | 0.9 | 0.54 | 0.92 | 0.59 | 1.06 | 5.57 | 2.78 | 0.03 | 0.05 | 0.18 | 0.08 | 0.04 | 0.14 | 0.08 | 0.12 | 0.03 | 0.08 | 0.06 | 0.08 |
| OTU1481 | 0.34 | 0.90 | 0.94 | 0.76 | 0.55 | 0.39 | 0.19 | 0.81 | 0.27 | 0.31 | 0.93 | 0.77 | 1.59 | 1.39 | 1.18 | 0.49 | 1.67 | 1.06 | 1.17 | 1.37 | 1.41 | 0.93 | 1.55 | 1.47 |
| OTU1985 | 0.03 | 0.06 | 0.01 | 0.03 | 0.05 | 0.03 | 0.1 | 0.09 | 0.14 | 0.03 | 0.01 | 0.16 | 2.4 | 2.26 | 1.77 | 1.4 | 1.58 | 0.96 | 1.46 | 2.02 | 2.67 | 1.13 | 2.05 | 1.73 |
| OTU1760 | 0.01 | 0 | 0 | 0 | 0.01 | 0 | 0.01 | 0 | 0 | 0.01 | 0 | 0 | 1.6 | 1.5 | 1.11 | 1.07 | 1.38 | 0.89 | 1.14 | 1.35 | 2.37 | 0.82 | 3.55 | 1.29 |
| OTU2356 | 0.48 | 1.76 | 0.98 | 1.5 | 1.37 | 3.33 | 0.25 | 1.01 | 1.13 | 0.79 | 1.62 | 0.85 | 0.05 | 0.03 | 0.01 | 0.07 | 0 | 0.04 | 0.16 | 0.04 | 0.02 | 0.05 | 0.01 | 0.06 |
| OTU893 | 1.32 | 2.17 | 1.98 | 0.69 | 1.06 | 0.21 | 0.09 | 0.46 | 0.18 | 0.14 | 0.17 | 0.25 | 0.23 | 0.17 | 0.43 | 0.31 | 0.4 | 2.76 | 0.11 | 0.11 | 0.06 | 0.08 | 0.38 | 0.61 |
| OTU207 | 0.37 | 0.33 | 0.4 | 0.5 | 0.43 | 0.65 | 0.18 | 0.31 | 0.18 | 0.27 | 0.49 | 0.34 | 0.79 | 0.87 | 0.84 | 0.2 | 0.46 | 0.69 | 0.66 | 1.05 | 1.01 | 0.94 | 1.19 | 1.13 |
| OTU1758 | 2.54 | 0.39 | 0.29 | 0.23 | 0.59 | 1.22 | 0.54 | 0.23 | 0.33 | 0.58 | 0.05 | 0.53 | 0.18 | 0.25 | 0.7 | 0.46 | 0.27 | 0.31 | 0.21 | 0.29 | 0.28 | 0.99 | 0.27 | 0.17 |
| OTU480 | 0.03 | 0.05 | 0.2 | 0.31 | 0.81 | 0.84 | 1.46 | 0.17 | 0.27 | 0.96 | 0.25 | 0.17 | 0.23 | 0.22 | 1.29 | 0.23 | 0.59 | 0.91 | 0.63 | 0.23 | 0.53 | 0.69 | 0.36 | 0.35 |
| OTU2139 | 0.01 | 0.06 | 0.05 | 0.23 | 0.2 | 0.38 | 0.1 | 0.15 | 0.49 | 0.23 | 0.03 | 0.12 | 0.29 | 0.34 | 1.03 | 0.58 | 1.3 | 1.02 | 1.08 | 0.42 | 0.64 | 0.48 | 1.05 | 0.77 |
| OTU1255 | 0.04 | 0.44 | 0.1 | 1.09 | 0.61 | 0.41 | 0.03 | 0.31 | 0.39 | 0.34 | 0.76 | 0.42 | 0.79 | 0.72 | 0.53 | 0.43 | 0.23 | 0.33 | 0.37 | 0.55 | 0.57 | 0.31 | 0.58 | 0.21 |
| OTU1696 | 0.06 | 0.08 | 0.74 | 0.74 | 0.24 | 0.16 | 5.14 | 0.13 | 0.02 | 0.02 | 1 | 0.42 | 0 | 0 | 0.06 | 0 | 0.45 | 0.02 | 0 | 0 | 0.03 | 0.01 | 1.11 | 0.04 |
| OTU1908 | 0.36 | 0.54 | 0.47 | 0.46 | 0.23 | 0.53 | 1.27 | 0.51 | 0.4 | 0.38 | 0.54 | 0.32 | 0.2 | 0.38 | 0.47 | 0.12 | 0.42 | 0.64 | 0.36 | 0.54 | 0.29 | 0.57 | 0.22 | 0.12 |
| OTU53 | 0.35 | 5.4 | 2.42 | 5.94 | 8.33 | 4.35 | 6.36 | 13.13 | 17.17 | 8.7 | 5.59 | 9.38 | 2.82 | 3.09 | 2.68 | 3.71 | 2.78 | 4.31 | 3.02 | 2.71 | 3.59 | 2.03 | 3.94 | 5.36 |
| OTU1211 | 0.21 | 2.25 | 1.09 | 2.09 | 3.34 | 2.32 | 3.56 | 4.42 | 4.9 | 7.2 | 1.94 | 3.70 | 2.06 | 1.96 | 1.97 | 2.43 | 2.17 | 2.34 | 2.05 | 1.76 | 3.30 | 1.82 | 4.76 | 3.13 |
| OTU668 | 3.07 | 1.54 | 3.63 | 4.04 | 4.33 | 4.93 | 10.02 | 1.74 | 2.15 | 13.32 | 1.78 | 2.30 | 0.26 | 0.31 | 0.68 | 0.29 | 0.66 | 4.18 | 0.77 | 0.51 | 0.39 | 0.94 | 0.6 | 0.92 |
| OTU750 | 0.01 | 0.58 | 0.15 | 0.29 | 0.2 | 0.02 | 0.05 | 1.13 | 0.58 | 0.14 | 0.04 | 1.09 | 4.75 | 4.32 | 1.02 | 2.72 | 2.94 | 0.23 | 3.08 | 2.69 | 2.81 | 1.31 | 1.72 | 0.4 |
| OTU613 | 0.04 | 1.11 | 0.29 | 0.83 | 0.7 | 0.46 | 0.68 | 2.31 | 2.31 | 0.54 | 0.43 | 1.56 | 2.23 | 2.15 | 1.22 | 2.11 | 1.33 | 0.59 | 1.65 | 1.73 | 1.66 | 1.33 | 1.56 | 0.77 |
| OTU1485 | 0.04 | 2.03 | 0.44 | 1.07 | 0.94 | 0.10 | 0.74 | 3.67 | 1.85 | 0.81 | 0.8 | 1.6 | 1.54 | 1.44 | 0.92 | 2.66 | 0.83 | 0.51 | 1.17 | 1.17 | 1.36 | 0.73 | 1.1 | 0.58 |
| OTU2376 | 0.39 | 0.59 | 0.51 | 0.39 | 0.57 | 0.25 | 2.79 | 1.76 | 0.83 | 1.22 | 0.92 | 1.24 | 0.96 | 0.85 | 1.02 | 1.31 | 1.74 | 1.25 | 1.84 | 0.71 | 1.14 | 0.73 | 2.41 | 1.02 |
| OTU2558 | 0.01 | 0.34 | 0.07 | 0.11 | 0.08 | 0.02 | 0.02 | 0.46 | 0.2 | 0.02 | 0.04 | 0.43 | 2.22 | 2.06 | 0.47 | 1.8 | 2.94 | 0.08 | 1.31 | 1.26 | 1.54 | 0.93 | 0.91 | 0.08 |
| OTU1519 | 0 | 0 | 0 | 0 | 0 | 0 | 0 | 0 | 0 | 0.01 | 0 | 0 | 1.39 | 1.06 | 1.89 | 6.3 | 0.17 | 0.04 | 0.57 | 0.54 | 2.14 | 0.35 | 0.18 | 0.04 |
| OTU1681 | 0.19 | 0.35 | 0.34 | 0.29 | 0.46 | 0.31 | 0.54 | 0.69 | 0.53 | 0.58 | 0.62 | 0.71 | 0.47 | 0.52 | 0.58 | 0.94 | 0.98 | 0.72 | 0.58 | 0.53 | 0.72 | 0.39 | 1.12 | 0.69 |
| OTU2000 | 0.01 | 0.48 | 0.21 | 0.52 | 0.33 | 0.09 | 0.11 | 1.65 | 1.74 | 0.24 | 0.25 | 1.03 | 0.76 | 0.59 | 0.3 | 0.91 | 0.96 | 0.18 | 0.65 | 0.56 | 0.75 | 0.4 | 0.8 | 0.24 |
| OTU1516 | 0.02 | 0.43 | 0.16 | 0.41 | 0.69 | 0.46 | 0.69 | 0.94 | 1.14 | 1.78 | 0.37 | 0.92 | 0.24 | 0.28 | 0.31 | 0.38 | 0.47 | 0.33 | 0.21 | 0.14 | 0.59 | 0.19 | 0.69 | 0.55 |

**Figure S1** Schematic representation of the different sampling sites in Dianchi Lake and Erhai Lake


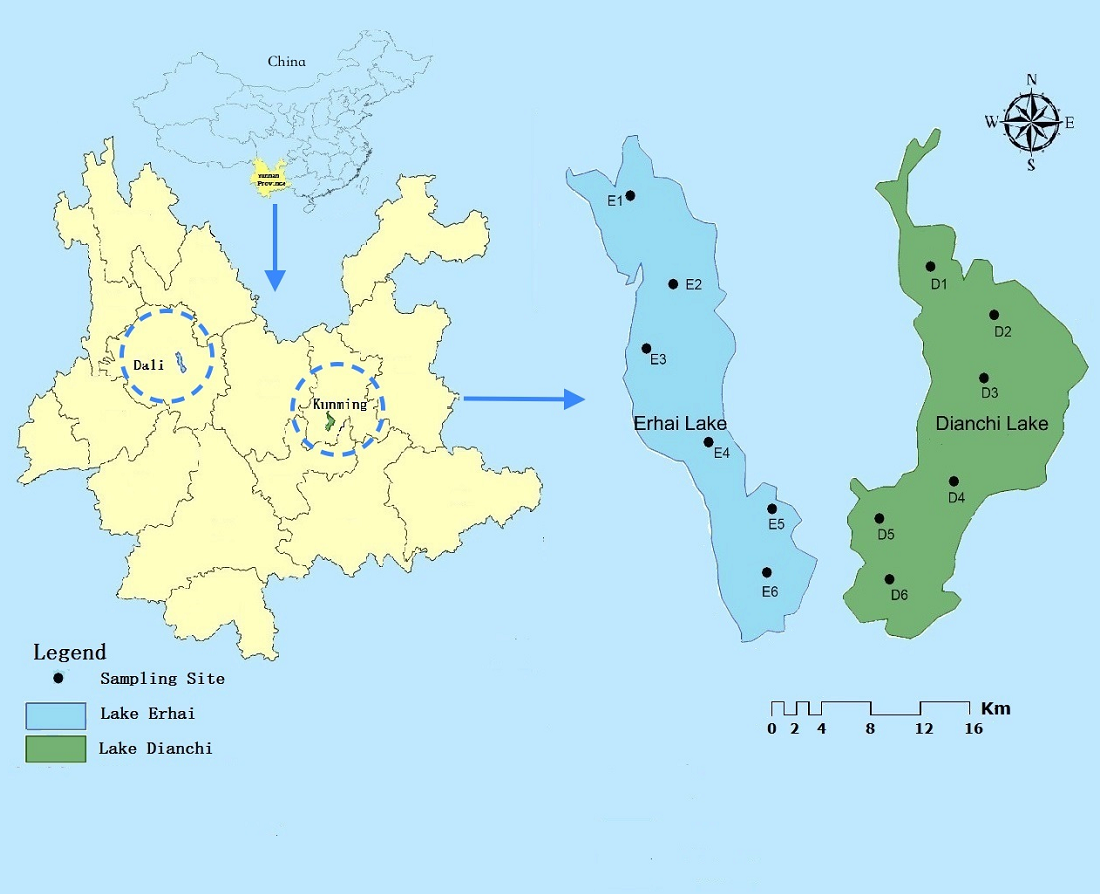


**Figure S2** Physicochemical features of water samples from Dianchi Lake and Erhai
Lake. Green and orange filled circles indicate the spring and summer water samples, respectively. *TN total nitrogen, TP total phosphorous, TOC total organic carbon, C/N the ratio of TOC to TN.*

**Figure S3** Physicochemical features of sediment samples from Dianchi Lake and Erhai Lake. Green and orange filled circles indicate the spring and summer sediment samples, respectively. *TN total nitrogen, TP total phosphorous, TOC total organic carbon, C/N the ratio of TOC to TN, ORP oxidation and reduction potential.*

the spring and summer waterent the spring and summer waterent the spring and summer waterent the spring and summer waterent the spring and summer waterent the spring and summer waterent the spring and summer waterent

**Figure S4** Comparison of the quantitative contribution of the sequences affiliated with different archaeal phyla to the total number of sequences from a given water or sediment sample. The archaeal sequences that could not be affiliated with known phylum are included as “others”. Samples DWAp1, DWAp2, DWAp3, DWAp4, DWAp5 and DWAp6 represent the April water samples from sites D1–D6 in Dianchi Lake, respectively. Samples DWAu1, DWAu2, DWAu3, DWAu4, DWAu5 and DWAu6 represent the August water samples from sites D1–D6 in Dianchi Lake, respectively. Samples EWAp1, EWAp2, EWAp3, EWAp4, EWAp5 and EWAp6 represent the April water samples from sites E1–E6 in Erhai Lake, respectively. Samples EWAu1, EWAu2, EWAu3, EWAu4, EWAu5 and EWAu6 represent the August water samples from sites E1–E6 in Erhai Lake, respectively. Samples DSAp1, DSAp2, DSAp3, DSAp4, DSAp5 and DSAp6 represent the April sediment samples from sites D1–D6 in Dianchi Lake, respectively. Samples DSAu1, DSAu2, DSAu3, DSAu4, DSAu5 and DSAu6 represent the August sediment samples from sites D1–D6 in Dianchi Lake, respectively. Samples ESAp1, ESAp2, ESAp3, ESAp4, ESAp5 and ESAp6 represent the April sediment samples from sites E1–E6 in Erhai Lake, respectively. Samples ESAu1, ESAu2, ESAu3, ESAu4, ESAu5 and ESAu6 represent the August sediment samples from sites E1–E6 in Erhai Lake, respectively.


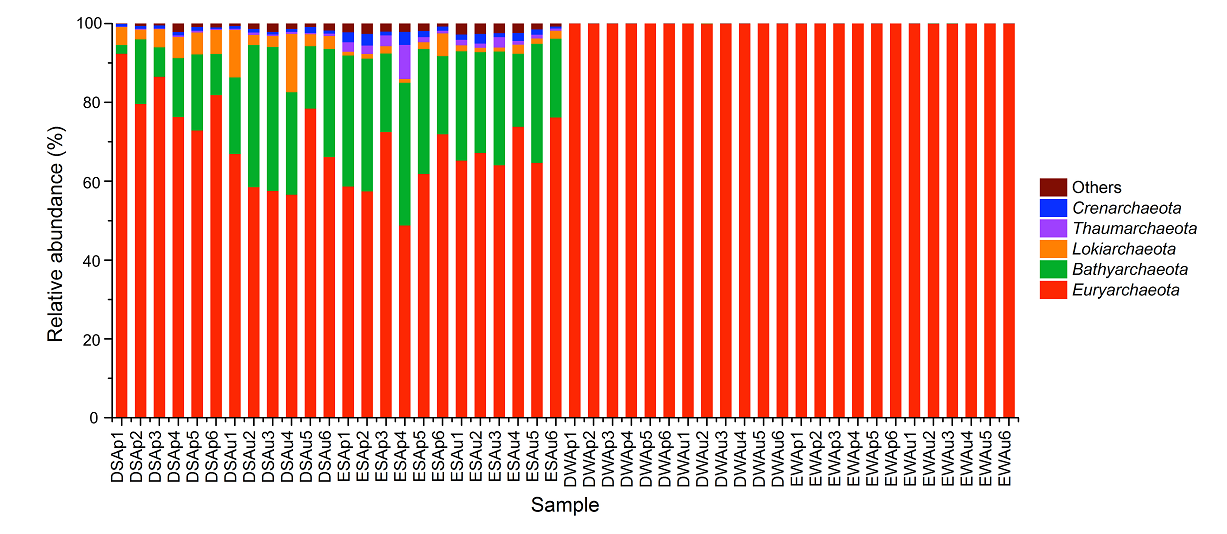


**Figure S5** Phylogenetic tree of the representative archaeal sequences and the reference sequences from GenBank. The number in parentheses represents the total number of the sequences in the same OTU. Numbers at the nodes indicate the levels of bootstrap support based on neighbor-joining analysis of 1,000 resampled datasets. The bar represents 5% sequence divergence.


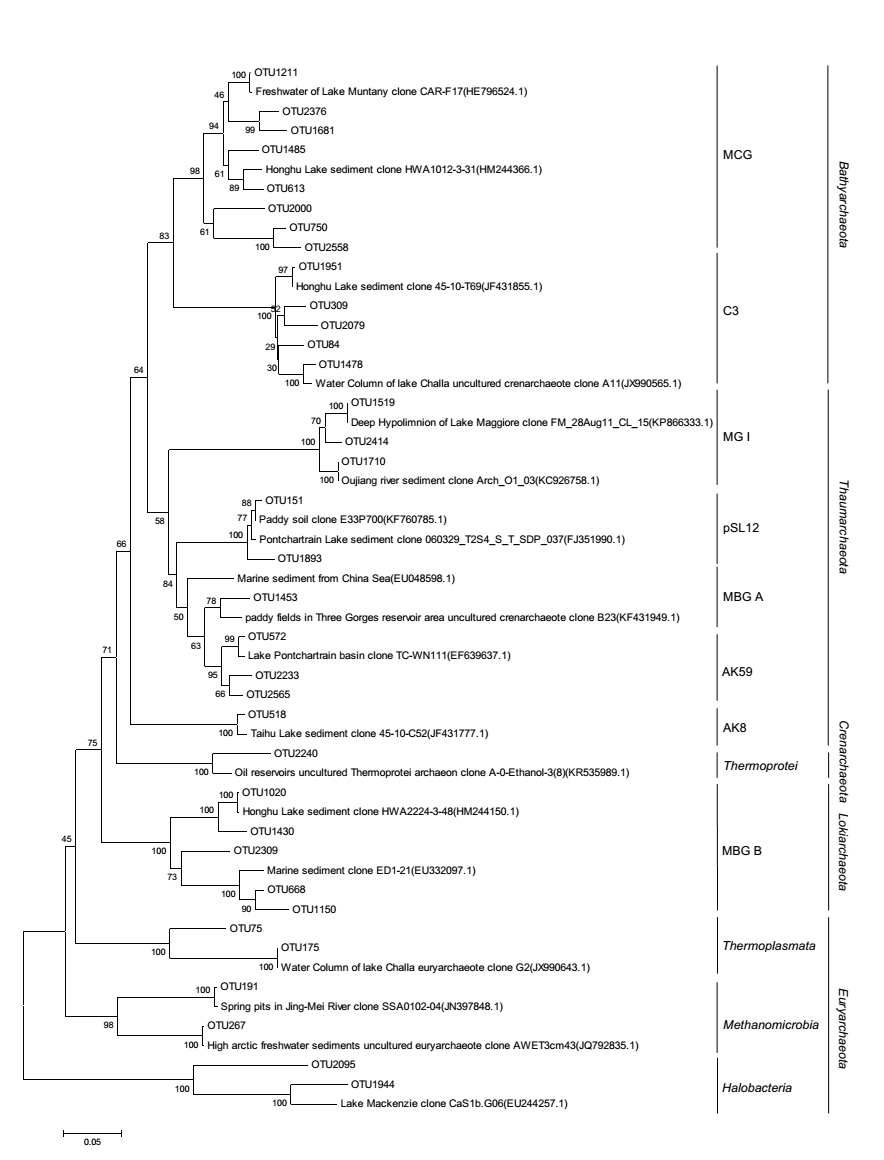

Supplement: Supplementary file 1 [file Data_Sheet_1.DOCX]
